# Biosynthetic Gene Clusters of *Astragalus* Triterpenoid Saponins: From Genome Discovery to Synthetic Biology and Biomanufacturing Prospects

**DOI:** 10.3390/plants15213466

**Published:** 2026-11-06

**Authors:** Tian Gao, Lei He, Yuan Wang, Shaofeng Su

**Affiliations:** 1 Inner Mongolia Academy of Agricultural & Animal Husbandry Sciences, Hohhot 010031, China; 18847603754@163.com (T.G.);; 2 College of Animal Science and Technology, Rongchang Campus, Southwest University, Chongqing 402460, China

**Keywords:** *Astragalus mongholicus*, Astragali Radix, astragaloside IV, cycloastragenol, triterpenoid saponin, biosynthetic gene cluster, synthetic biology, heterologous production

## Abstract

Astragaloside IV is the best-characterized cycloartane-type triterpenoid saponin and the pharmacopoeial quality marker of Astragali Radix, with documented cardioprotective, antitumor, and immunomodulatory effects. Supply is constrained by long cultivation cycles that vary with production practice, replant disease, and phytochemical variability. Six representative genome assemblies are summarized for the genus, and they show that triterpenoid tailoring genes are partly, but not universally, clustered: a ~4 Mb biosynthetic gene cluster houses the oxidative and glycosylating enzymes, whereas the backbone synthase gene *AmOSC3* lies outside it. The core route from 2,3-oxidosqualene to astragaloside IV has been reconstituted enzyme by enzyme; the in planta downstream routing, the acetylation steps forming astragalosides I and II, and the regulation, compartmentalization, transport, and storage of these saponins remain unresolved. Heterologous production is at the proof-of-concept stage: 1.04 mg/L cycloastragenol in yeast (versus the 25 g/L artemisinic acid precedent), 2.224 mg astragaloside IV/g dry weight in *Nicotiana benthamiana*, and a 4.2-fold total-astragaloside increase in *AmUGT15*-overexpressing hairy roots. This review consolidates genome mining, pathway biochemistry, and synthetic-biology engineering; benchmarks current platforms against pharmacopoeial quality requirements; and maps the bottlenecks between proof of concept and sustainable biomanufacturing.

## 1. Introduction: From Traditional Medicine to Genome-Driven Drug Discovery

Astragali Radix (Huangqi)—the dried root of *Astragalus mongholicus* Bunge (syn. *A. membranaceus* var. *mongholicus* (Bunge) P.K.Hsiao) and, under the Flora of China treatment, *A. membranaceus* Fisch. ex Bunge—ranks among the foremost qi-tonifying herbs in the Chinese Pharmacopoeia. Its pharmacology rests on three compound classes: triterpenoid saponins, isoflavones, and polysaccharides [1,2]. The cycloartane-type saponins, centered on astragaloside IV, function as both official quality markers and the most heavily studied bioactive constituents, with anti-inflammatory, antioxidant, antifibrotic, immunomodulatory, cardioprotective, and antitumor activities documented in preclinical models [3,4,5,6,7,8,9,10]. Two pharmacological facts bear directly on production strategy. First, astragaloside IV has a measured absolute oral bioavailability of 2.2% in rats (20 mg/kg oral versus 2.5 mg/kg intravenous) and 7.4% in beagle dogs, a limitation of the molecule rather than of its source [1,11,12]; its metabolism (25 catalogued metabolites), drug-interaction profile, and formulation strategies such as mixed micelles have been characterized accordingly [13,14,15]. Second, saponin content varies with genotype, region, and harvest timing, making field-grown material chemically inconsistent [16,17,18,19,20]. Cycloastragenol, the aglycone, has attracted attention for anti-aging properties through telomerase activation, the NRF2/ARE pathway, and macrophage-polarization modulation [21,22,23], and semi-synthetic derivatives outperform the parent compound in telomerase activation [21,24]. Separately, a randomized, double-blind, placebo-controlled trial investigated an *Astragalus*-based supplement [25]. It should be noted that astragaloside IV is only one of more than a hundred cycloartane- and oleanane-type saponins in the root, and the efficacy of the herb may involve multiple constituents and their interactions [1,2]; this review therefore covers the biosynthesis of the astragaloside family as a whole, with astragaloside IV as the best-resolved branch. These properties make the saponins attractive drug leads but also expose raw-material supply as a serious industrial bottleneck, and pharmacokinetics-guided quality-marker frameworks have been proposed for *Astragalus* evaluation [26].

Taxonomic note. Nomenclature in this review follows Plants of the World Online (POWO, Royal Botanic Gardens, Kew; accessed 2026) [27], which accepts *Astragalus mongholicus* Bunge and treats *A. membranaceus* var. *mongholicus* (Bunge) P.K.Hsiao as a homotypic synonym and *A. membranaceus* Fisch. ex Bunge as a heterotypic synonym; Flora of China, by contrast, maintains *A. membranaceus* as a distinct accepted species [28]. The source germplasm of the cited genomic and biochemical studies maps onto this treatment as follows: the reference genome of Chen et al. [29] was generated from material described as *A. membranaceus* var. *mongholicus*, whereas the assemblies of Fan et al. [30] and Qin et al. [31] and the pathway studies of Xu et al. [32], Zhang et al. [33], Chen et al. [34], and Duan et al. [35] used cultivated material described as ‘*A. membranaceus*’, i.e., Astragali Radix germplasm within the *A. mongholicus*–*A. membranaceus* complex. Differences reported between these assemblies are therefore described here as assembly- or germplasm-level variation rather than assumed species-level divergence, pending voucher-based identification under a consistent taxonomic framework, and the herbal drug is referred to as Astragali Radix (Huangqi), distinct from the genus name *Astragalus*.

Supply problems originate in agronomy. A single cropping cycle takes 3 to 5 years from sowing to harvest. Under wild-simulated cultivation, three-year-old plants give the best astragaloside IV content and return on investment [36]. Replant failure under continuous cropping has several causes. Monoculture allows rhizosphere pathogens such as *Fusarium avenaceum*, *F. flocciferum*, and related species to accumulate and cause recurrent root rot [37,38,39]. Volatile allelochemicals in root exudates, including (E,E)-2,4-decadienal and (E)-2-hexenal, inhibit germination and seedling growth in a dose-dependent manner [40]. Soil microbial diversity drops in parallel, destabilizing the rhizosphere community [41]. Crop rotation helps but does not resolve the problem [42,43]. Active-compound content also varies widely across regions and harvest timing [16,17]. Maxent models have quantified how altitude, temperature, and precipitation influence astragaloside IV levels [16]. Expanding acreage alone therefore cannot deliver a stable, uniform feedstock. Quality control spans the full chain from field to harvest [18,26], and metabolic profiling can separate production areas and cultivation methods [19,20]. Even *A. mongholicus* and *A. membranaceus* differ detectably in saponin composition [19].

Synthetic biology offers an alternative approach: transferring the plant pathway into a microbial or plant chassis and fermenting the target compound rather than extracting it. Artemisinin and ginsenosides have already been produced in this way. For *Astragalus*, two prerequisites had to be met first: a high-quality genome and an enzyme-level map of the core pathway. Both arrived within two years. The *A. mongholicus* chromosome-level genome was published in 2023 [29], the *A. membranaceus* assembly in 2024 [30], and total biosynthesis of astragalosides was reported by Xu et al. in Nature Plants that same year [32]. *Astragalus* triterpenoid saponins thus became accessible to heterologous production.

Rationale, gap, and objectives. Genomic and biochemical studies published between 2022 and 2025 have transformed this field, but the evidence is scattered across genome papers, enzyme-characterization studies, and engineering reports that use inconsistent nomenclature and non-comparable production metrics, and several pathway models remain in conflict. This review aims to (i) consolidate the genomic resources and biosynthetic gene cluster (BGC) organization of *Astragalus*; (ii) critically synthesize the elucidated pathway from 2,3-oxidosqualene to astragaloside IV, distinguishing demonstrated steps from inferred ones; (iii) compare heterologous and plant-based production platforms on a transparent quantitative basis; and (iv) define the biological and engineering bottlenecks that separate proof of concept from sustainable biomanufacturing.

### Literature Search and Review Methodology

This is a narrative review; to make its coverage transparent we document our search strategy. PubMed, Web of Science, Scopus, and Google Scholar were searched for literature published from January 2000 to 2026 (PubMed: publication dates 1 January 2000 to 1 August 2026; Web of Science and Scopus: publication years 2000–2026, cross-checked in October 2026). To ensure that the search adequately captured literature across the four thematic pillars of this review, the strategy combined four thematic blocks: (i) genomics (Astragalus in combination with genome, genomic, transcriptome, or whole genome); (ii) biosynthesis and biosynthetic gene clusters (Astragalus in combination with triterpenoid, triterpene, saponin, astragaloside, or cycloastragenol, and with biosynthesis, biosynthetic gene cluster, gene cluster, cytochrome P450, glycosyltransferase, oxidosqualene cyclase, or pathway); (iii) regulation (Astragalus in combination with triterpenoid, triterpene, saponin, or astragaloside, and with regulation, transcription factor, epigenetics, chromatin, or gene expression); and (iv) biotechnological production (Astragalus, astragaloside, or cycloastragenol in combination with synthetic biology, metabolic engineering, heterologous expression, heterologous production, de novo biosynthesis, hairy root, microbial production, cell factory, or yeast). The complete search strings, as run in PubMed and adapted to the syntax of Web of Science and Scopus, are provided in Supplementary File S1; Google Scholar was screened with the same term combinations to identify additional relevant literature. English- and Chinese-language publications were included. The four thematic blocks retrieved 182, 298, 163, and 58 records in PubMed, respectively (592 unique records after merging), together with 1139 records in Web of Science and 1764 records in Scopus (per-block counts in Supplementary File S1). Inclusion criteria: peer-reviewed primary studies and reviews addressing *Astragalus* genomics, triterpenoid saponin biosynthesis and regulation, or heterologous/cell-culture production; pharmacological and clinical studies were included only where needed to motivate the production rationale. Reference lists of included papers were screened for additional sources. Studies were excluded if they (i) were not peer-reviewed publications (e.g., conference abstracts, editorials, or theses); (ii) had no discernible relevance to any of the four thematic blocks or to the background sections; (iii) reported purely pharmacological or clinical effects of astragaloside IV or cycloastragenol without biosynthetic, genomic, regulatory, or production content; or (iv) were duplicate or superseded reports of the same data.

Records were screened in two stages: titles and abstracts were first screened against the inclusion and exclusion criteria, and the surviving records were then assessed for eligibility. Titles and abstracts were screened independently by two authors (T.G. and L.H.), and disagreements were resolved by discussion with S.S. Of the 592 unique PubMed records, 90 were excluded at the title/abstract stage (89 as off-topic and 1 as a non-peer-reviewed item), and a further 42 were excluded at the eligibility stage because they contained no biosynthetic, genomic, regulatory, or production content (e.g., purely pharmacological or clinical studies of astragaloside IV), leaving 460 eligible records. Records retrieved from Web of Science and Scopus were deduplicated against this set and screened with the same criteria. All eligible records were catalogued in a shared reference library, from which studies were selected for citation on the basis of originality, data quality, and topical relevance. Backward citation chaining of key reviews and landmark studies was used to capture the comparative literature on plant triterpenoid biosynthetic gene clusters and synthetic biology in other species, as well as background pharmacology and agronomy; this procedure, together with the reference-list screening described above, yielded the 114 sources cited in this review. The search captured all of the key primary studies underpinning Section 2, Section 3 and Section 4, including both chromosome-level *Astragalus* genomes, the biosynthetic gene cluster and pathway-elucidation studies, and the heterologous-production studies.

## 2. Genomes and Biosynthetic Gene Clusters: The Genetic Basis of *Astragalus* Triterpenoid Diversity

Dissecting any plant specialized-metabolic pathway begins with a high-quality genome. Without knowing where genes are located on chromosomes and how they are arranged, it is impossible to determine which genes cooperate or which duplication events drove metabolic diversification. *Astragalus* genomics provides this foundation.

### 2.1. Genome Sequencing and Assembly of Astragalus

Six representative genome assemblies are summarized for the genus (Table 1). A chromosome-scale reference for the medicinal complex was the *A. mongholicus* genome published by Chen et al. in 2023 [29]. The 1.47 Gb assembly combined PacBio CCS long reads with Hi-C scaffolding, reaching a contig N50 of 9.79 Mb and a scaffold N50 of 181.02 Mb. Nearly all of the sequence (99.75%) was anchored to nine pseudochromosomes, with 27,868 protein-coding genes predicted and BUSCO completeness at 96.84%. Earlier work by Yin et al. used multi-platform full-length transcriptome sequencing to search for isoflavone and triterpenoid saponin biosynthetic genes in *A. mongholicus* [44], laying groundwork for the genome era. Fan et al. then published the *A. membranaceus* genome one year later [30]: 1.43 Gb, contig N50 of 1.67 Mb, also assigned to nine pseudochromosomes, with 29,914 predicted protein-coding genes. The independent ONT-based *A. membranaceus* assembly of Xu et al. [32] (AM-ONT) comprises 1.44 Gb anchored to nine chromosomes, with contig/scaffold N50 values of 2.82/184.69 Mb and 38,398 predicted protein-coding genes. Genome sizes and chromosome numbers are similar among these earlier medicinal assemblies, consistent with within-genus conservation, but differences in gene counts and repeat proportions require interpretation in light of assembly and annotation methods. A burst of LTR retrotransposon expansion roughly 0.13 million years ago in *A. mongholicus*—dated from LTR sequence divergence, although the LTR-specific substitution rate was not specified in the reported methods—may have contributed to its somewhat larger genome [29]. In 2025, Qin et al. published a gap-free telomere-to-telomere (T2T) *A. membranaceus* assembly (‘AM-T2T’; 1.39 Gb; scaffold N50 180.45 Mb; BUSCO 99.63%; QV 57.51; 32,600 high-confidence genes, including 248 CYP450s and 163 UGTs), resolving 158.58 Mb of previously unassembled sequence and enabling the detection of 1413 structural variants relative to the *A. mongholicus* assembly [31]. Notably, the T2T assembly resolved eight chromosomes (16 telomeres and eight centromeres), whereas Fan et al. anchored their assembly to nine pseudochromosomes; this discrepancy remains to be reconciled. Beyond the medicinal lineage, the 595.52 Mb chromosome-scale genome of the green-manure species *A. sinicus* (2n = 16; 34,253 predicted genes; BUSCO 91.10%) [45] and the haplotype-resolved, chromosome-scale genome of the arctic-alpine *A. alpinus* (766.58 Mb for haplotype 1; eight chromosomes) [46] provide outgroup resources for comparative and synteny analyses within the genus. Table 1 summarizes the key metrics of these six assemblies.

### 2.2. Gene Family Expansion and Tandem Duplication

Because predicted gene numbers are sensitive to assembly continuity and annotation pipelines, expansion claims here rest on the orthogroup-level comparison of the primary study rather than on absolute family sizes. Chen et al. [29] combined AUGUSTUS/SNAP predictions, GeMoMa homology evidence, and transcript-derived models using EvidenceModeler/PASA; orthogroups were inferred with OrthoFinder v2.4 and DIAMOND (E-value ≤ 10^−3^). CAFE v4.2 tested expansion against reconstructed ancestral family sizes, requiring both family-wide and Viterbi *p* values < 0.05. Of 218 gene families identified as significantly expanded in *A. mongholicus* relative to the other legumes included in that orthogroup analysis, 93 (42.7%) included tandem duplications [29]. Tandem duplication is a well-known evolutionary mechanism for specialized-metabolic diversification across plants, documented in caffeine and crocin pathways [47], tomato root specialized-metabolic BGCs [48], and many medicinal plant genomes [49,50,51]. The annotated enzyme complements are large: the CYP450 family comprises 243 members, with 109 forming 39 clusters on eight chromosomes, and the UGT family comprises 154 members, 97 of them clustered; the T2T annotation reports comparable numbers (248 CYP450s and 163 UGTs) [31]. A directly reported legume benchmark is 154 UGTs in *A. mongholicus* versus 115 in *A. sinicus*; whether differences among independently annotated medicinal assemblies reflect true lineage-specific expansion or annotation differences will require standardized re-annotation with a single pipeline [29]. The biologically relevant point is functional. CYP450s and UGTs are the two enzyme classes that oxidize and glycosylate the triterpenoid backbone, so additional copies provide candidates for the enzymatic variety behind astragaloside structural diversity, whose specific contributions require functional validation. *Astragalus* has long occupied arid, high-UV, nutrient-poor environments. The elaboration of specialized metabolic pathways may reflect adaptation to environmental stress [29].

### 2.3. Discovery and Characterization of Biosynthetic Gene Clusters

Biosynthetic gene clusters (BGCs) were another key discovery. In plants, BGCs are physically contiguous, functionally coordinated gene sets on a chromosome that feed into the same metabolic pathway; they typically span 35 kb to several hundred kb and contain 3 to 15 genes, although exceptionally large clusters spanning several megabases have recently been reported [32,52]. plantiSMASH analysis of *A. mongholicus* detected 24 secondary-metabolism clusters, three of them related to triterpenoid biosynthesis [29]. Cluster I holds three β-amyrin synthase (bAS) genes, which serve oleanane-type triterpenoids. Cluster II carries a single lupeol synthase (LUS) gene. Cluster III is particularly notable. Its annotation contains one lanosterol synthase (LAS), two cycloartenol synthase (CAS) genes, and two LUS genes, and its OSC genes lie in close physical proximity to CYP450 and UGT genes on chromosome 7. In the related species *A. sinicus*, the corresponding OSC genes lack these neighboring modifiers [29]. This comparison identifies candidate physical linkage between backbone and tailoring genes; its effects on pathway efficiency and co-regulation remain to be established. Physically close genes may share open chromatin states and transcription factors, but this remains untested for *Astragalus* cluster III. Cluster III spans Chr07:104,786,544–105,263,544 bp (477,001 bp from the first annotated gene start to the last gene end) and contains Am07G013590, Am07G013600, Am07G013610, Am07G013620, Am07G013630 (LAS), Am07G013640 (CYP88), Am07G013650 (UGT73), Am07G013660 (CAS), Am07G013670, Am07G013680 (CAS), Am07G013690 (LUS), and Am07G013700 (LUS); the first four genes and Am07G013670 are annotated as other genes (Supplemental Table 31 of [29]). Heterologous assays verified cycloartenol production for Am07G013660 and lupeol production for Am07G013690, whereas Am07G013680 did not yield detectable cycloartenol [29].

Notably, the clustered architectures differ between the two sequenced medicinal assemblies. In *A. mongholicus*, cluster III physically associates OSC genes (LAS, CAS, and LUS) with neighboring CYP450 and UGT modifiers on chromosome 7 [29], whereas the approximately 4 Mb cluster in *A. membranaceus* contains only tailoring enzymes, with the backbone synthase *AmOSC3* located outside the cluster [32]. This contrast indicates that, even within a single genus, coordinated triterpenoid biosynthesis can be achieved with or without physical linkage to the backbone synthase, and argues against a universal assembly rule for *Astragalus* BGCs. Whether the two arrangements reflect germplasm variation, assembly and annotation differences, or different stages of cluster evolution remains an open question that comparative synteny analysis across additional *Astragalus* genomes could resolve.

### 2.4. Evolutionary Context and Epigenetic Regulation of BGCs

*Astragalus* BGCs sit within a broader research landscape on plant triterpenoid clusters. The *Arabidopsis* thalianol cluster is reshuffled by chromosomal inversion and related events, and organizational differences across species yield species-specific triterpenoids [53]; metabolic gene clusters also vary widely among *Arabidopsis* ecotypes [54]. *Aralia elata* generates triterpenoid diversity through tandem duplication of CYP72A and UGT73 genes combined with dammarenediol synthase exon loss, and yeast co-expression has enabled de novo synthesis of 13 aralosides [55]. In Solanaceae, the withanolide BGC gave rise to tissue-specific subclusters through subfunctionalization [56]. Horse chestnut (*Aesculus*) carries two triterpenoid BGCs formed by whole-genome duplication that drive aescin biosynthesis [57]. Plant BGCs are not universal, however. Cárdenas et al. noted that triterpenoid biosynthetic genes can be clustered or dispersed depending on lineage, producing a mosaic evolutionary pattern [58]. Smit and Lichman emphasized that BGCs are comparatively rare between species and span a wide size range, with their occurrence tied to particular lineages and pathways [59]. The absence of a cluster III counterpart in *A. sinicus* indicates that the cluster is lineage-restricted rather than genus-wide: this specific annotated architecture has so far been reported in the Chen et al. medicinal assembly, and its association with high cycloartane accumulation remains correlational. Resolving when and how cluster III arose will require microsynteny comparison of at least the *A. mongholicus*, *A. membranaceus* (AM-T2T), and *A. sinicus* assemblies—ideally extended to *A. alpinus*—within a phylogenetic framework. The clustered organization nevertheless simplifies genome mining: physically adjacent genes are more likely to participate in the same pathway, so candidate enzymes can be shortlisted rapidly from BGCs [52,60], and yeast platforms have been used to systematically characterize enzyme functions within plant BGCs [61]. These examples are cited for their specific relevance to *Astragalus*: tandem duplication contributes to the *Astragalus* enzyme repertoires, while inversion in *Arabidopsis* provides a comparative hypothesis for cluster evolution, and the *Arabidopsis* and Solanaceae cases define the chromatin-level questions that the following paragraph poses for cluster III.

Whether clustered genes share epigenetic regulation has received little attention. In the Solanaceae withanolide cluster, chromatin-state differences between subclusters lead to tissue-specific expression [56], and activation of the *Arabidopsis* thalianol cluster coincides with local chromatin decompaction [53]. *Astragalus* cluster III spans approximately 477 kb in the Chen et al. assembly [29]. Whether its OSC, CYP450, and UGT members share an open-chromatin domain or are coordinated by a common set of transcription factors is unknown. The cited study does not report cluster-specific chromatin conformation or ChIP-seq data [29]. Answering this question would clarify the functional logic of BGCs and guide promoter selection and co-expression design for heterologous synthesis. Bulk-tissue and developmental-stage transcriptome data from *A. mongholicus* [29] provide expression evidence for candidate cluster III genes, but whether apparent co-expression reflects co-regulation driven by physical proximity or shared cis-elements requires further experimental dissection. The situation is further complicated by the likelihood that regulatory mechanisms differ from cluster to cluster, with species and pathways potentially relying on distinct co-expression strategies [52,59].

## 3. Pathway Elucidation: From 2,3-Oxidosqualene to Astragaloside IV

**Figure 1:**
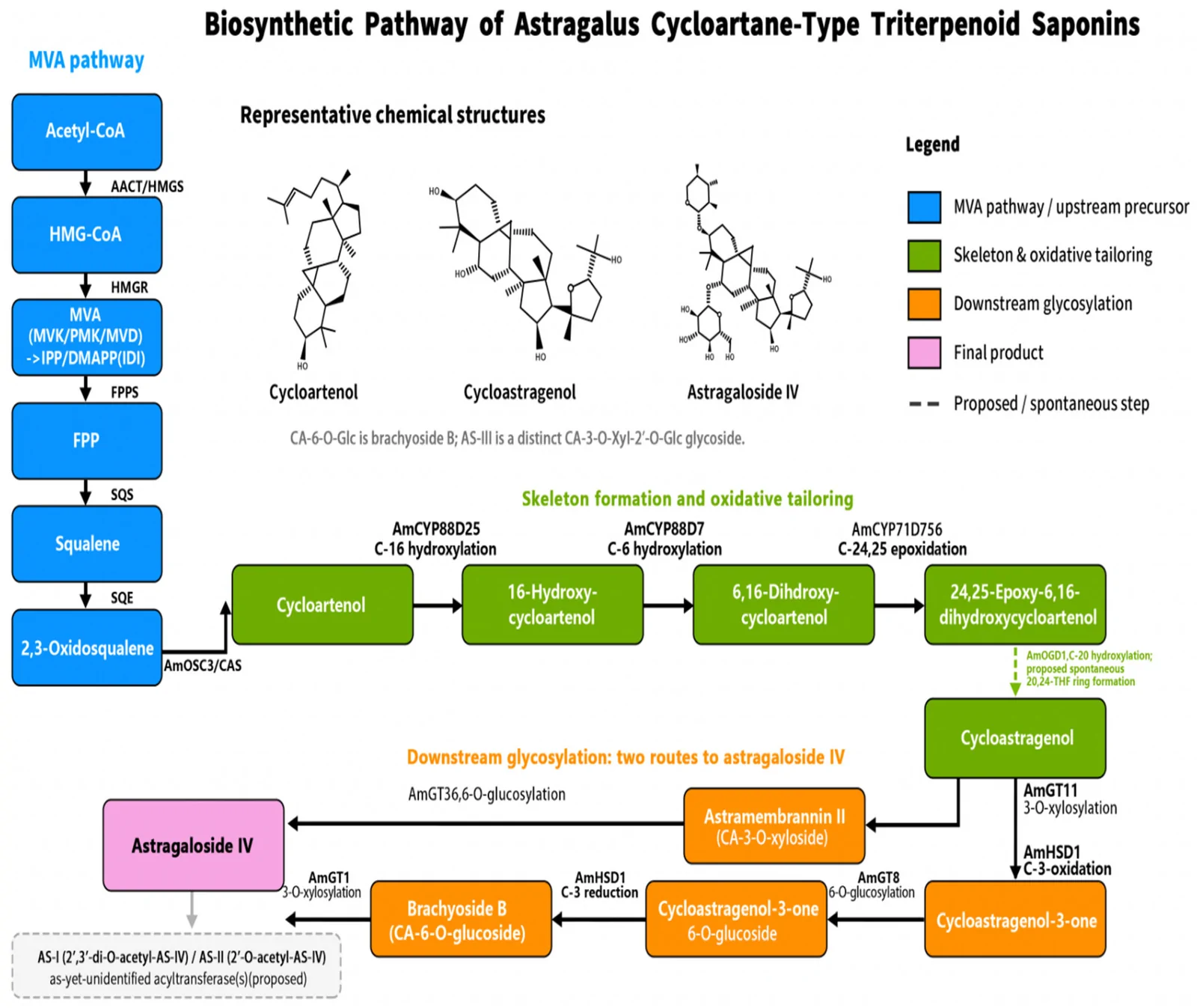
Biosynthetic pathway of *Astragalus* cycloartane-type triterpenoid saponins. The MVA pathway (blue) supplies 2,3-oxidosqualene; cycloartenol synthase (AmOSC3/CAS, green) builds the cycloartane backbone. Oxidative tailoring proceeds through three CYP450 steps—AmCYP88D25 (C-16 hydroxylation), AmCYP88D7 (C-6 hydroxylation), and AmCYP71D756 (C-24,25 epoxidation)—and the 2-oxoglutarate-dependent dioxygenase AmOGD1 (C-20 hydroxylation), followed by proposed spontaneous formation of the 20,24-tetrahydrofuran ring to yield cycloastragenol; named intermediates are 16-hydroxycycloartenol, 6,16-dihydroxycycloartenol, and 24,25-epoxy-6,16-dihydroxycycloartenol. Downstream (orange), the C-3 oxidation-reduction route (AmHSD1, AmGT8, AmGT1) and the direct route (AmGT36, AmGT11) yield astragaloside IV (pink); astragalosides I and II are 2′,3′-di-O-acetylated and 2′-O-acetylated derivatives of astragaloside IV, formed by an as-yet-unidentified acyltransferase (dashed box). Astragaloside III carries a 2′-O-glucosylated 3-O-xylosyl chain and is distinct from brachyoside B, the cycloastragenol 6-O-glucoside [32,34,35]. Enzyme identities are experimentally demonstrated [32,33,34,35]; the depicted oxidation order follows the intermediate evidence of [32], and a strictly sequential scheme versus a metabolic grid remains unresolved (see text). Solid arrows denote experimentally demonstrated conversions; dashed arrows and the dashed box denote proposed steps (the spontaneous 20,24-tetrahydrofuran ring formation and the as-yet-unidentified acyltransferase).

The genome provides a candidate gene inventory. Pathway elucidation determines how these genes assemble into a functional biosynthetic route. *Astragalus* triterpenoid saponin biosynthesis breaks down into three stages: upstream precursor generation, backbone assembly with oxidative tailoring, and downstream glycosylation. With the core route mapped in 2024, each step from 2,3-oxidosqualene to astragaloside IV has been reconstituted, although the downstream routing in planta remains unresolved (Figure 1).

### 3.1. Upstream Precursor Supply: The MVA Pathway

The upstream mevalonate (MVA) pathway is highly conserved across plants. It starts from acetyl-CoA and proceeds through AACT and HMGS to HMG-CoA; the rate-limiting enzyme HMGR reduces HMG-CoA to mevalonate, which is converted by subsequent MVA-pathway enzymes to IPP and DMAPP. These condense into farnesyl diphosphate (FPP), which squalene synthase (SQS) and squalene epoxidase (SQE) convert to 2,3-oxidosqualene [29]. This portion of the pathway shows no species specificity, but MVA pathway genes are strongly expressed in *A. mongholicus* roots [29], providing the precursor flux that underpins root-specific triterpenoid saponin accumulation. That *Astragalus* active compounds concentrate in roots has long been observed; the transcriptome data now provide a molecular explanation. Regulation of the plant MVA pathway involves multiple feedback loops. When sterols are in excess, HMGR is degraded through the ER-associated degradation pathway [62]. SQS supplies squalene to both sterol and specialized-triterpenoid pathways; competing OSCs determine how 2,3-oxidosqualene is routed to different backbones. In heterologous systems, relieving these feedback constraints to push precursor flux toward triterpenoids is a central theme in metabolic engineering and is discussed in detail below.

### 3.2. Backbone Formation: OSC Cyclization

Triterpenoid-backbone diversification begins at 2,3-oxidosqualene cyclization. The oxidosqualene cyclase (OSC) family determines the triterpenoid backbone: CAS produces cycloartenol (the cycloartane backbone), bAS produces β-amyrin (oleanane-type), LAS produces lanosterol, and LUS produces lupeol [29]. OSC catalytic diversity and the control of substrate conformation have been studied systematically in several plants [63,64]. Because *Astragalus* triterpenoid saponins are predominantly cycloartane-type, CAS is the critical initiating enzyme. Chen et al. identified multiple OSCs from *A. membranaceus* and demonstrated that the conserved VFM/VFN motif is essential for catalytic function; modification of this motif substantially increased cycloartane-type triterpenoid yields [65]. Duan et al. subsequently functionally characterized cycloartenol synthase alongside four glycosyltransferases, confirming the central role of CAS in cycloastragenol-type saponin biosynthesis [35]. Two CAS genes in the *A. mongholicus* genome are highly expressed in roots during vegetative growth [29], consistent with the spatiotemporal pattern of astragaloside accumulation. The catalytic mechanism of OSCs is also noteworthy. Cyclization of 2,3-oxidosqualene is governed jointly by substrate conformation and active-site geometry; the same substrate can yield different backbones through different carbocation intermediates depending on the OSC [58,65]. When CAS produces cycloartenol, it retains the 9β,19-cyclopropane ring. This ring distinguishes cycloartane-type triterpenoids from other tetracyclic triterpenoids and may influence the regioselectivity of subsequent CYP450 oxidations.

### 3.3. Oxidative Modification: CYP450 and Non-CYP450 Oxidases

Several oxidation steps separate cycloartenol from cycloastragenol. CYP450s are heme-thiolate monooxygenases that catalyze regioselective oxidation of the triterpenoid backbone and are the core enzyme family driving specialized-metabolic diversification in plants [66,67]. At least 149 plant triterpenoid CYP450s have been functionally characterized, spanning subfamilies that include CYP716, CYP88D, and CYP72A [66]. Wang et al. identified 21 AmP450s in *A. mongholicus* as likely participants in triterpenoid saponin biosynthesis. Co-expression analysis revealed tissue- and stage-dependent expression patterns, with several genes positively correlated with triterpenoid accumulation [67]. In eudicots the CYP716 family dominates triterpenoid diversification [64,66], but oxidative tailoring of cycloartane-type triterpenoids in *Astragalus* does not rely on CYP716. It instead involves the CYP88D and CYP71D subfamilies and a non-CYP450 oxidase. Xu et al.’s total biosynthesis study in Nature Plants identified every oxidation step from cycloartenol to cycloastragenol [32]. Three CYP450s (AmCYP88D25, AmCYP88D7, and AmCYP71D756) catalyze C-16 hydroxylation, C-6 hydroxylation, and C-24,25 epoxidation, respectively. A 2-oxoglutarate-dependent dioxygenase, AmOGD1, catalyzes C-20 hydroxylation. The C-20 hydroxyl and C-24,25 epoxide are then proposed to react spontaneously to form the 20,24-tetrahydrofuran ring, the characteristic side-chain structure of cycloastragenol—a cyclization inferred from the characterized intermediates that has not been demonstrated in vitro, so an enzyme-catalysed alternative cannot be excluded [32]. The participation of AmOGD1 was unexpected. Oxidative modification of plant triterpenoid backbones had been considered almost exclusively a CYP450 function, yet AmOGD1 belongs to the 2-oxoglutarate-dependent dioxygenase family [68,69,70]. Plants can apparently recruit different oxidase lineages to modify triterpenoid backbones [32]. 2-oxoglutarate-dependent dioxygenases are non-heme iron enzymes that use 2-oxoglutarate as a cosubstrate and Fe^2+^ as a cofactor and drive substrate hydroxylation through oxidative decarboxylation. In plants they catalyze diverse oxidations of flavonoids, alkaloids, and gibberellins [68,70], but their participation in triterpenoid backbone modification had rarely been reported. This result broadens the known oxidase repertoire of plant specialized metabolism [69]. CYP88D7 had already appeared in Wang et al.’s co-expression analysis as a root-enriched candidate significantly correlated with astragaloside IV content [67], consistent with Xu et al.’s functional characterization and showing that genomic-era co-expression analysis can generate productive leads for pathway elucidation.

### 3.4. C-3 Oxidation-Reduction and Glycosylation: The Four-Step Pathway

The gap between cycloastragenol and astragaloside IV was closed by two studies published in 2024 that revealed an unexpected reaction logic. In The Plant Journal, Zhang et al. described a four-step pathway [33]. In step 1, the hydroxysteroid dehydrogenase AmHSD1 oxidizes the C-3 hydroxyl of cycloastragenol to a ketone, producing cycloastragenol-3-one. In step 2, the glycosyltransferase AmGT8 glucosylates at C-6. In step 3, AmHSD1 acts again, this time reducing the C-3 ketone back to a hydroxyl. In step 4, AmGT1 xylosylates at C-3, yielding astragaloside IV. The key feature of this pathway is the bifunctionality of AmHSD1: a single enzyme catalyzing both oxidation and reduction, two seemingly contradictory reactions. The “detour” may reflect AmGT8 acceptor recognition: in the demonstrated route, the C-3 hydroxyl is first converted to a ketone and restored after 6-O-glucosylation [33]. A specific steric-hindrance mechanism remains to be established, and direct glycosylation is possible with other enzymes [32]. A similar oxidation-glycosylation-reduction sequence occurs in α-tomatine biosynthesis in tomato [33], suggesting a conserved plant strategy for glycosylating sterically shielded hydroxyl groups. Mechanistically, AmHSD1 belongs to the short-chain dehydrogenase/reductase superfamily, and its catalytic direction depends on the cofactor redox state and substrate availability. In planta, the redox environment of different compartments may influence whether AmHSD1 acts as an oxidase in step 1 or a reductase in step 3, although the specific compartmentalization mechanism and cofactor cycling remain unclear. This bifunctionality is both an advantage in heterologous expression (one enzyme, two reactions) and a challenge, demanding precise control of catalytic direction.

Glycosylation diversifies saponin structures and is a major determinant of structural diversity and biological activity. Chen et al. systematically characterized and protein-engineered glycosyltransferases from *A. membranaceus*, building a combinatorial UGT system (AmGT1/5/8/9) that synthesized 13 cycloartane-type saponins with conversion rates of 22.6% to 98.7% [34]. AmGT1 exhibited donor promiscuity, accepting multiple sugar donors including UDP-glucose and UDP-xylose, and could even form novel ginsenosides [34]. Rational mutations such as AmGT1 G146V/S and AmGT8 A394F altered UGT regioselectivity and expanded the saponin structural space [34]. Donor promiscuity and regioselectivity of UGTs are core drivers of structural variety in plant glycosylated natural products [71,72]. In plant family 1 UGTs, sugar-donor specificity is set by key residues in the PSPG motif [73], and rational engineering can shift donor preference [73,74]. The expanded UGT family in *Astragalus* (154 members [29]) is a rich enzymatic reservoir for this diversity. Systematic UGT mining in medicinal plants such as *Panax notoginseng* [74] and *Platycodon grandiflorus* [75] provides methodological templates for further *Astragalus* UGT research. At the structural level, UGT regioselectivity is governed by acceptor-binding-site residues in the active pocket. The G146 position of AmGT1 lies near the sugar-donor binding region; the G146V/S mutations alter UDP-glucose/UDP-xylose selectivity and reduce C-25 glycosylation activity [34]. The A394F mutation of AmGT8 likely restricts substrate orientation by introducing a bulky aromatic side chain, enabling 6-O-glucosylation in place of the 2′-O-glucosylation used for wild-type AmGT8 in the combinatorial system [34]. These engineering efforts extend the accessible saponin structural space and provide models for understanding the structural basis of UGT regioselectivity.

It is noteworthy that the two 2024 studies reached astragaloside IV through different downstream routes. Zhang et al. demonstrated a four-step route in which AmHSD1-mediated C-3 oxidation precedes AmGT8-catalyzed 6-O-glucosylation, followed by C-3 reduction and AmGT1-catalyzed 3-O-xylosylation [33]. Xu et al., by contrast, reconstituted astragaloside IV in *N. benthamiana* using AmGT36 and AmGT11 without an HSD-mediated detour [32]. AmGT11 directly xylosylates cycloastragenol at C-3, while AmGT36 glucosylates astramembrannin II at C-6 and also accepts cycloastragenol to form brachyoside B [32]. The relative contribution of these routes in planta remains unresolved; Zhang et al. themselves noted that an efficient 6-O-glycosyltransferase acting directly on cycloastragenol cannot be excluded [33]. Clarifying which route operates in *Astragalus* roots, and why, should be a priority, because it defines the minimal gene set required for heterologous production.

### 3.5. Total Biosynthesis Validation and Pathway Integration

Integrating these findings, Xu et al. achieved total biosynthesis of astragalosides in Nature Plants [32]. Analysis of the *A. membranaceus* genome led them to a triterpenoid biosynthetic gene cluster spanning approximately 4 Mb, the largest plant natural-product BGC reported to date at the time of writing. Unlike previously known triterpenoid BGCs, this cluster contains no backbone synthase (OSC). It houses only oxidative modification enzymes (AmCYP88D25, AmCYP88D7, AmCYP71D756, AmOGD1) and glycosyltransferases (AmGT11, AmGT36); the OSC gene (*AmOSC3*) lies outside the cluster [32]. This organization suggests that the assembly logic of plant BGCs may be more flexible than previously recognized. Physical proximity between backbone synthase and modifier enzymes is not a prerequisite for functional coordination. The team confirmed the roles of six cluster genes through in vitro enzyme activity assays, *N. benthamiana* transient expression, yeast in vivo functional analysis, and hairy-root RNAi mutant characterization. By co-expressing these genes with *AtCPR1*, *AmOSC3*, and *NbtHMGR* in *N. benthamiana*, they produced astragaloside IV in tobacco leaves at 2.224 mg/g dry weight, the AG IV-specific endpoint [32]. From the genomic cluster to the fully reconstituted biochemical pathway, the core biosynthetic route of *Astragalus* cycloartane saponins is now established. This should not be read as a complete solution: the relative contribution of the two downstream routes in planta, the acyltransferase(s) forming astragalosides I and II, the transcriptional and chromatin-level regulation of the clusters, enzyme compartmentalization and cofactor cycling, and the transport, storage, and secretion of the products all remain open (Section 5.4). Table 2 summarizes the key enzymes identified to date, and Table 3 reconciles the gene names used for the downstream glycosylation routes.

### 3.6. Biosynthetic Evidence for Other Astragalus Saponins

Beyond the astragaloside IV branch, gene-level evidence for the more than one hundred other *Astragalus* saponins remains fragmentary. For cycloartane-type homologues, Duan et al. functionally characterized a cycloartenol synthase and four glycosyltransferases that glycosylate the cycloastragenol scaffold [35], and Chen et al. extended this enzymatic toolbox into a combinatorial UGT system that generated 13 cycloartane-type saponins in vitro [34]; overexpression of *AmUGT15* in hairy roots raised total astragaloside content 4.2-fold, although the compound-level products of this activity remain to be resolved [76]. For the oleanane-type branch, the cluster I bAS genes supply the β-amyrin backbone [29], but no tailoring CYP450 or UGT acting on *Astragalus* oleanane saponins has been characterized to date. Several steps on known homologues are likewise unassigned: the glucosyltransferase extending the astragaloside III sugar chain and the acyltransferase(s) forming astragalosides I and II remain unidentified (Section 3.4; Figure 1). Gene-level coverage is therefore centered on astragaloside IV, and completing the enzymatic map of the remaining homologues is a prerequisite for evaluating—and eventually reproducing—the multi-component composition of the herbal drug.

## 4. Synthetic Biology: From Pathway Validation to Heterologous Production

**Figure 2:**
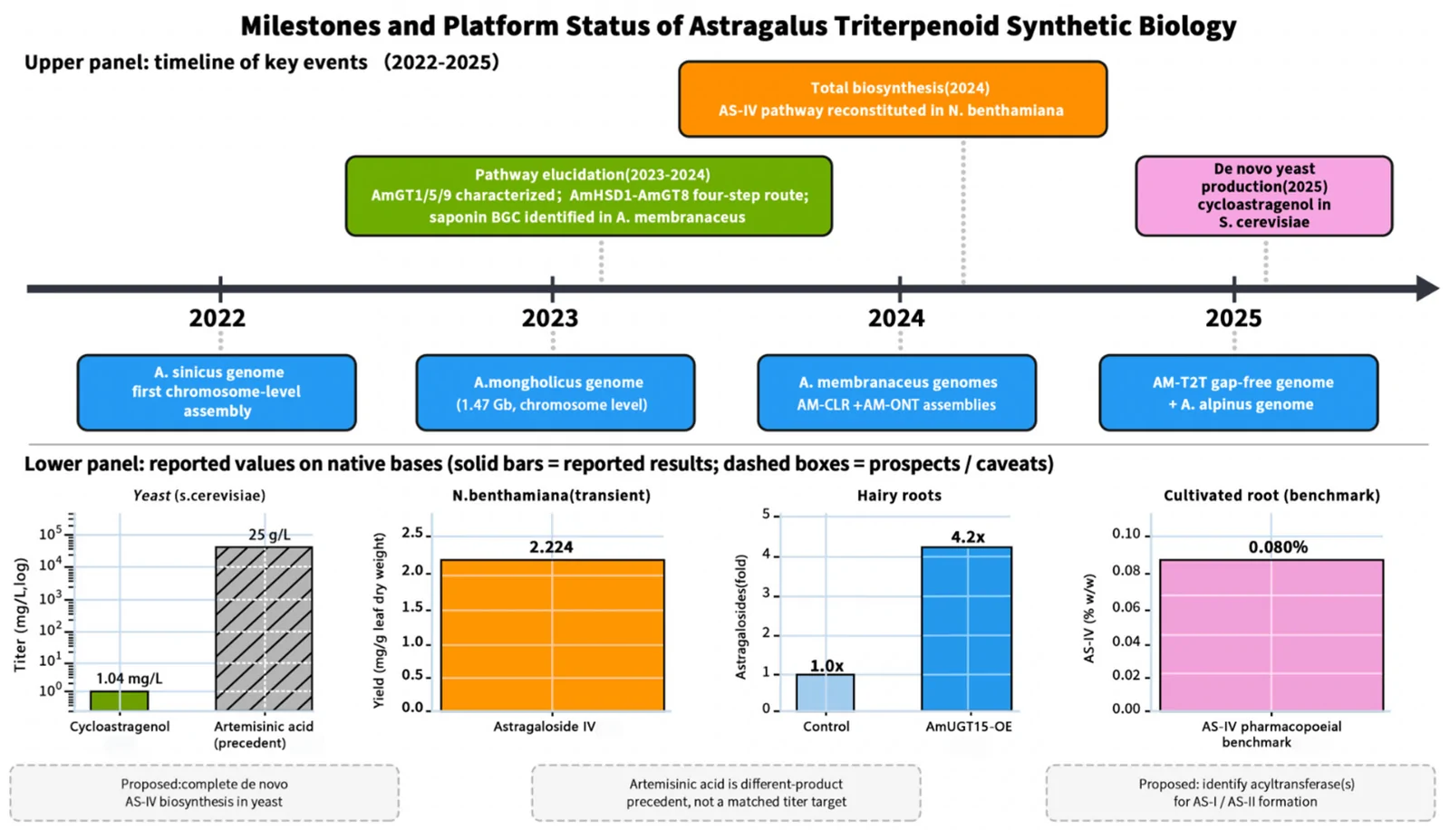
Milestones and platform status of *Astragalus* triterpenoid synthetic biology. Upper panel: timeline of key events (genome assemblies, 2022–2025; pathway elucidation, 2023–2024; total biosynthesis, 2024; de novo yeast production, 2025). Lower panel: reported production values on their native bases (solid bars, experimentally demonstrated; dashed boxes, proposed strategies): 1.04 mg/L cycloastragenol in yeast [77] versus the 25 g/L artemisinic acid precedent [78]; 2.224 mg astragaloside IV/g dry weight in *N. benthamiana* [32]; 4.2-fold total-astragaloside enhancement in *AmUGT15*-overexpressing hairy roots [76]; pharmacopoeial assay specification of 0.080% (*w*/*w*) astragaloside IV in dried root [79,80]. Box colors in the upper panel group the milestones thematically (blue, genome assemblies; green, pathway elucidation; orange, total biosynthesis; pink, de novo yeast production); bar colors in the lower panel distinguish the platforms (green, yeast cycloastragenol; hatched gray, artemisinic acid precedent; orange, *N. benthamiana* astragaloside IV; light and dark blue, hairy-root control and the *AmUGT15*-overexpressing line, respectively; pink, pharmacopoeial benchmark).

Synthetic biology became feasible once the core pathway had been mapped. Relocating a plant metabolic route of more than a dozen enzymes, spanning multiple cellular compartments, into a heterologous chassis is more complex than introducing individual genes in isolation. Two approaches have emerged. One deploys *Saccharomyces cerevisiae* as a microbial fermentation chassis; the other relies on *N. benthamiana* for plant transient expression. Each has distinct strengths and bottlenecks (Figure 2).

### 4.1. Precursor Supply Engineering

On the yeast track, the main hurdle is precursor supply. All triterpenoid backbones derive from squalene, and triterpenoid output is ultimately capped by squalene availability. Squalene overproduction in yeast is a well-developed metabolic-engineering paradigm. Expressing a truncated HMGR to escape feedback inhibition raises MVA flux [81]. Reconstituting the MVA pathway in mitochondria achieves compartmentalized isolation and curbs competition with sterol synthesis [82,83]. Machine learning identified mevalonate kinase Erg12p as a key bottleneck enzyme, and the resulting platform strain delivered a 94-fold increase in terpenoid production [84]. Downregulation of ERG7 (lanosterol synthase) stabilizes ERG1 (squalene epoxidase) and channels squalene toward 2,3-oxidosqualene, pushing β-amyrin production to 4216.6 mg/L [85]. In combination, these strategies have driven squalene titers to 55.8 g/L in *S. cerevisiae* and 51.2 g/L in *Yarrowia lipolytica* [86], providing ample precursor pools for downstream triterpenoid work. Metabolic recycling of storage lipids can further bolster squalene accumulation [87]. High squalene accumulation does not automatically translate into high triterpenoid titers, however. Squalene epoxidation to 2,3-oxidosqualene is catalyzed by ERG1, which supplies 2,3-oxidosqualene to both endogenous sterol synthesis and heterologous triterpenoid pathways; endogenous ERG7 competes with heterologous OSCs for this substrate. Du et al. expanded 2,3-oxidosqualene supply by downregulating ERG7 to improve ERG1 protein stability [85], but whether this approach remains effective after introducing heterologous OSCs depends on the relative activity of the introduced OSC and endogenous ERG7 on 2,3-oxidosqualene. In most yeast engineering for triterpenoid synthesis, conversion efficiency from 2,3-oxidosqualene to the target triterpenoid falls well short of what squalene levels would predict. This gap is a major yield bottleneck [85,86].

### 4.2. De Novo Synthesis of Cycloastragenol in Yeast

Zhang et al. achieved de novo cycloastragenol synthesis in yeast following precursor-pathway engineering [77]. They reconstituted the MVA pathway in yeast peroxisomes, accumulating squalene to 656.55 mg/L, then introduced *AmCAS1* and the four oxidases identified by Xu et al. (AmCYP88D25, AmCYP88D7, AmCYP71D756, AmOGD1). The result was 1.04 mg/L cycloastragenol, a demonstration of de novo cycloastragenol production in yeast. Feasibility was established, but the titer remains low and requires process-specific economic evaluation. Because the squalene titer (656.55 mg/L) and the cycloastragenol titer originate from successive stages of the same engineering programme rather than from a single matched cultivation, their ratio cannot be read as a conversion efficiency; it is only an unmatched arithmetic comparison: 1.04 mg/L cycloastragenol corresponds to roughly 0.13 mol% of 656.55 mg/L squalene (mass ratio 0.16%), which does not quantify conversion efficiency or precursor-carbon loss. Matched intermediate quantification or isotope-labelling experiments will be needed to identify flux limitations involving 2,3-oxidosqualene supply, CAS catalytic efficiency, and oxidase co-expression imbalance. Three factors likely contribute: insufficient 2,3-oxidosqualene supply, low CAS catalytic efficiency, and imbalanced co-expression of the four oxidases in yeast. Byun et al. found that overexpressing the transcription factor Rap1 boosts yeast triterpenoid saponin production by enhancing precursor supply and heterologous gene expression; ginsenoside CK yield increased 4.5-fold [88]. This points to a new optimization avenue, though its effectiveness in the *Astragalus* triterpenoid system remains untested.

### 4.3. CYP450 Heterologous Expression Bottlenecks and Optimization

Plant CYP450s are ER-anchored membrane proteins, and during pathway reconstruction they often suffer steep activity losses in yeast due to unsuitable membrane environments, CPR (cytochrome P450 reductase) mismatch, or inefficient protein folding [89]. The *Astragalus* pathway involves three CYP450s (AmCYP88D25, AmCYP88D7, AmCYP71D756) and one non-CYP450 oxidase (AmOGD1). CYP450 heterologous expression is a major bottleneck in triterpenoid synthetic biology. As a plant chassis, *N. benthamiana* offers a native ER environment and post-translational modification capacity, and it has become an important platform for heterologous plant natural-product synthesis [90,91]. Zhang et al. co-expressed AmHSD1, AmGT8, and AmGT1 in *N. benthamiana* transient expression and successfully produced astragaloside IV [33]. Xu et al. reconstructed the complete route from 2,3-oxidosqualene to astragaloside IV in *N. benthamiana*, reaching 2.224 mg/g dry weight [32]. The limitation of a plant chassis is the difficulty of scaled fermentation. It is better suited to rapid pathway validation and complex saponin assembly, while yeast carries the main industrial expectations. Optimizing CYP450-CPR pairing is a standard strategy for improving CYP450 activity in yeast. Comparative analysis of legume CPRs showed that homologous pairing (CYP716A with CPR-I, CYP72A with CPR-II) gives the best results [92], and different CPR choices can produce a 4.5-fold difference in oleanolic acid yield [93]. Xu et al. used *Arabidopsis*-derived AtCPR1 as the electron-transfer partner for CYP450s in *N. benthamiana* [32], but which CPR pairs best with *Astragalus* CYP450s in yeast awaits systematic screening. Cofactor-coupled CYP450 engineering has enabled high-level asiatic acid production [94], and regioselective oxidation by the CYP450-CPR bi-enzymatic system has been characterized in *Ilex* [95]. Modifying the yeast ER membrane to increase CYP450 anchoring sites, or using fusion-protein strategies to bring CYP450 and CPR into closer spatial proximity, has proven effective in other triterpenoid systems. AmOGD1, a 2-oxoglutarate-dependent dioxygenase, does not depend on CPR for catalysis and may express more readily in yeast than CYP450s, but ensuring intracellular supply of the cosubstrate 2-oxoglutarate and cofactor Fe^2+^ remains an open task. These are the observed and potential bottlenecks; the corresponding engineering remedies—CPR screening, ER remodelling, directed evolution, and fusion constructs—are evaluated together in Section 5.3.

### 4.4. Glycosyl Donor Supply

Another bottleneck is glycosyl donor supply. Astragaloside IV synthesis requires both UDP-glucose and UDP-xylose. In yeast, UDP-glucose is largely consumed by cell-wall biosynthesis and glycogen metabolism, which can constrain the pool available for heterologous UGTs. Overexpression of UGP1 can partially relieve UDP-glucose shortage [96], but UDP-xylose synthesis in yeast is more involved and requires introduction of heterologous UDP-glucose dehydrogenase (UGD) and UDP-xylose synthase (UXS), converting UDP-glucose through UDP-glucuronic acid to UDP-xylose [75]. Saponin hydrophobicity may affect interactions with cell membranes; effects on host growth and product yield [82,86] require direct testing, and extracellular product secretion remains unsolved. In in vitro enzyme catalysis systems, glycosyl donor supply can be controlled because UDP-sugars can be added exogenously. This explains why Chen et al. achieved efficient synthesis of 13 saponins in vitro, with conversion rates of 22.6% to 98.7% [34]. In living cells, however, intracellular UDP-sugar pools are contested by multiple metabolic routes, and simply overexpressing synthases is often insufficient to expand the donor pool meaningfully. Matched UDP-glucose-pathway tests in [97] yielded 44.62 and 39.72 mg/L calycosin-7-O-glucoside versus 47.60 mg/L in control strain PP01, but optimization strategies for UDP-xylose remain immature.

### 4.5. Lessons from Other Triterpenoid Synthetic Biology

Successes in other triterpenoid synthetic biology provide lessons for *Astragalus*. Semi-synthesis of artemisinin is a landmark in microbial production of plant natural products: Paddon et al. reached 25 g/L artemisinic acid in *S. cerevisiae* [78], building on the work of artemisinin discoverer Tu Youyou [98]. Yeast synthesis of ginsenosides has also advanced substantially. Wang et al. achieved efficient ginsenoside Rh2 synthesis by optimizing UGT and CYP450 expression [99]. Compartmentalization engineering can raise lipophilic saponin titers [100], and protopanaxatriol-type saponins have reached high-level sustainable production in engineered yeast [101]. Gram-scale diosgenin production [102] and combinatorial biosynthesis of saikosaponins [103] and betulinic acid [104] all confirm that plant triterpenoid pathways can be transplanted into microbial chassis. Pathway elucidation for triterpenoid saponins in *Prunella vulgaris* [105] and *Saponaria vaccaria* [106] also offers comparative references for *Astragalus* pathway optimization. The specific relevance of these examples to *Astragalus* is direct: each demonstrates one of the engineering levers—precursor supply, UGT/CYP450 balancing, compartmentalization, or combinatorial glycosylation—evaluated for the astragaloside pathway in Section 4.1, Section 4.2, Section 4.3, Section 4.4 and Section 5.3.

### 4.6. Technology Platforms and Isoflavone Synthetic Biology

CRISPR-Cas9 genome editing is a powerful tool for microbial metabolic engineering. It enables multi-site simultaneous editing, scarless deletion, and precise insertion, and has been used in plant-derived terpenoid production to tune MVA pathway gene copy numbers and knock out competing pathways [107]. Automated biofoundries couple the design-build-test-learn (DBTL) cycle with robotic automation, enabling thousands of yeast transformations and screenings per week and markedly accelerating pathway optimization [108]. Deployment of these platforms should compress the timeline from laboratory validation to industrialization for *Astragalus* triterpenoid synthetic biology.

Isoflavones, another class of *Astragalus* active compounds, have also been produced synthetically, offering a reference for glycosylated-compound biomanufacturing. Chen et al. and Zhang et al. independently achieved de novo calycosin-7-O-glucoside synthesis in yeast by introducing calycosin pathway enzymes, with reported titers of 0.22 mg/L and 47.6 mg/L, respectively [96,97] (Table 4).

## 5. Sustainable Production: Challenges, Complementarity, and Future Paths

Synthetic biology of *Astragalus* triterpenoid saponins remains at an early stage on the path from laboratory validation to industrial production. The cycloastragenol titer of 1.04 mg/L remains a proof-of-concept endpoint [77]. Astragaloside IV has reached 2.224 mg/g dry weight in *N. benthamiana* transient expression [32], but de novo astragaloside IV synthesis from simple carbon substrates in yeast has not been demonstrated in the cited studies. Closing this gap requires breakthroughs on several fronts.

### 5.1. Sustainability Benchmarking

Two reference points anchor the sustainability discussion. First, the Chinese Pharmacopoeia requires Astragali Radix to contain not less than 0.080% (*w*/*w*) astragaloside IV, i.e., 0.8 g per kg of dried root [79,80]. This is an assay-defined quality limit rather than a crop yield; sample treatment can convert other astragalosides to astragaloside IV [80]. Second, an established microbial plant-natural-product precedent, artemisinic acid, reached 25 g/L in engineered yeast [78]; as an unmatched comparison between different products, the current cycloastragenol titer of 1.04 mg/L [77] is roughly four orders of magnitude lower, and de novo astragaloside IV synthesis from simple carbon substrates in yeast has not been demonstrated in the cited studies. Table 5 summarizes reported platform endpoints; comparable resource inventories are unavailable: field cultivation is established but requires land, water, and long cultivation periods; yeast fermentation requires nutrient feedstock and shortens the cycle to days but is currently limited by titer and by solvent-intensive downstream purification [109]; hairy-root culture retains the plant’s metabolic background with cycle times of weeks [76] but faces bioreactor scale-up constraints; transient plant expression offers speed and complex-molecule competence with scale-dependent productivity still to be assessed [91]; and semi-synthesis has gram- and kilogram-scale demonstrations [110,111] and remains feedstock-dependent. Because credible cost-per-gram estimates are not possible for laboratory-stage platforms, no quantified economic claims are made, and ‘sustainable production’ is framed as the field’s objective rather than a demonstrated outcome.

### 5.2. Semi-Synthesis and Plant Cell/Tissue Culture Routes

Two production routes additional to de novo synthesis deserve explicit treatment. Cycloastragenol can be produced by semi-synthesis: total *Astragalus* saponins are extracted from cultivated roots and deglycosylated, by acid hydrolysis or enzymatically, to the aglycone. Reported examples include 96.5% AS-IV-to-cycloastragenol conversion at gram scale [110] and a 2.54-kg chemical cycloastragenol preparation [111]. This feedstock-dependent route inherits the feedstock problems of field cultivation and requires solvent-intensive purification. Adventitious-root culture of *A. membranaceus*, demonstrated in a 5-L bioreactor with 3.4 mg total saponins/g dry weight [113], offers root-like saponin profiles without field cultivation, with cycle times of weeks; cell-suspension cultures have also been investigated, although comparable product-specific yield and stability data were not established here [114]. Relative to these routes, de novo yeast production [77] and transient plant expression [32,33] remain laboratory-stage: they trade maturity for independence from plant feedstock. Table 5 compares the five listed routes on yield, duration, scale, downstream-processing requirements, and economic maturity; their relative economic performance and future supply contribution require process-specific evaluation.

### 5.3. Engineering Strategies for Oxidase Catalytic Efficiency

Building on the observed bottlenecks documented in Section 4.3, the pathway requires three CYP450s plus the dioxygenase AmOGD1, and low CYP450 activity in yeast is a recurring problem in triterpenoid synthetic biology. Several options exist. The first is screening yeast-compatible CPRs for *Astragalus* CYP450s or introducing plant CPRs; the AtCPR1 used by Xu et al. in *N. benthamiana* could serve as a starting point for yeast expression [32]. Others include remodeling the ER membrane to increase CYP450 anchoring sites and applying directed evolution to improve CYP450 stability and catalytic efficiency [89]. Balanced expression of pathway enzymes matters just as much. From MVA to astragaloside IV, over a dozen enzymes participate, and overexpression or insufficiency at any step can cause intermediate accumulation, toxicity, or flux leakage. Fine-tuned regulation through promoter engineering, copy-number optimization, and multi-enzyme fusion is therefore required [77]. Multi-enzyme fusion links two or more enzymes via flexible linkers, enabling intermediate transfer between enzymes in close proximity and reducing diffusion loss. In triterpenoid synthesis, CYP450–CPR fusion constructs, which generate self-sufficient monooxygenases, have been used to boost catalytic efficiency (OSCs are not cytochrome P450s and do not require CPR) [89,95]. Whether AmHSD1-UGT fusion is viable, however, depends on the substrate-product compatibility and spatial orientation of the two reaction steps, and no experimental validation has been reported. As discussed in Section 4.3, AmOGD1 escapes the CPR and ER-membrane constraints of CYP450s; its yeast-specific challenge instead lies in securing intracellular 2-oxoglutarate and Fe^2+^ supply, which has not been specifically optimized.

### 5.4. Biological Bottlenecks Beyond Enzyme Catalysis

Pathway completion does not by itself deliver production. Four biological layers remain largely unexplored in *Astragalus*. (1) Regulation: jasmonate signalling controls triterpenoid saponin pathways in several plants, and methyl-jasmonate elicitation was the key to pathway elucidation in *Saponaria vaccaria* [106]; whether cluster III or the 4 Mb cluster is jasmonate-responsive, and which transcription factors mediate co-expression, is unknown. (2) Transport and storage: plants typically sequester saponins in vacuoles or export them via ABC/MATE transporters; no astragaloside transporter has been identified, although product localisation will determine both accumulation capacity in planta and extraction strategy in cell factories. (3) Toxicity and self-resistance: saponins integrate into membranes and can disrupt membrane function, a potential host constraint that requires testing for astragaloside production; how *Astragalus* roots tolerate gram-per-kilogram saponin loads—and whether that tolerance can be transferred to a chassis—is unstudied. (4) Cofactor and donor supply: UDP-xylose formation in yeast requires heterologous UDP-glucose dehydrogenase (UGD) and UDP-xylose synthase (UXS) activities [75], and AmOGD1 adds 2-oxoglutarate and Fe^2+^ requirements that have not been specifically optimised (Section 4.4); the UDP-xylose limitation is currently inferred rather than measured for this pathway—calycosin-7-glucoside titers of 0.22 and 47.6 mg/L came from independent studies [96,97], and matched UDP-glucose-pathway tests in [97] did not improve the PP01 control endpoint, but UDP-sugar pool sizes and UXS/UGD-engineering titer changes were not reported for the *Astragalus* triterpenoid reconstruction systems cited here, a specific gap identified here. Each of these layers maps onto a concrete engineering strategy, and progress will likely require addressing them jointly rather than enzyme by enzyme.

### 5.5. Complementarity Between Synthetic Biology and Traditional Cultivation

Synthetic biology and traditional cultivation are complementary rather than substitutive. For industrial applications that demand high-purity single compounds such as astragaloside IV as an API, yeast cell factories offer short production cycles, freedom from seasonal and geographic constraints, and the potential for controlled product quality, subject to analytical validation [86]. Whole *Astragalus* extracts contain multiple compound classes, so substitution by a single biomanufactured compound requires separate pharmacological assessment; traditional cultivation and standardized farming remain important supply routes. The two routes will coexist over the long term, serving different scenarios. Genomic information also strengthens traditional breeding. Molecular markers linked to active-compound content can be derived from genomic variation to accelerate elite-variety selection [29,30], and mining genes tied to continuous-cropping obstacles can furnish targets for breeding resistant varieties [39,41]. The 218 expanded gene families identified in the *A. mongholicus* genome [29] include triterpenoid and flavonoid biosynthesis genes alongside disease- and stress-resistance genes. Pairing synthetic-biology approaches with traditional breeding, for example using genomic selection markers to breed high-saponin, cropping-obstacle-resistant varieties, could be a practical route to raising overall industry efficiency.

Whether biomanufactured astragaloside IV can substitute for the herbal drug depends on the product concept. As a single-compound active pharmaceutical ingredient, comparability requires demonstration: structural identity (including stereochemistry) is established by NMR and HR-MS against the pharmacopoeial reference standard, purity and impurity profiles by validated HPLC methods, and pharmacological comparability in appropriate functional assays; pharmacokinetic bioequivalence requires dedicated exposure studies; its low oral bioavailability is a property of the molecule, not of its origin, and is addressed by formulation rather than by the production route. Equivalence to the whole herb cannot be claimed for any single compound, because the efficacy of Astragali Radix may involve multiple components and their interactions (Section 1); defined multi-component mixtures are conceivable through combinatorial UGT synthesis [34] or co-culture strategies, but their pharmacological parity would require dedicated demonstration. Equivalence claims are therefore restricted to the single-compound context.

### 5.6. Hairy Root Culture

Hairy root culture has emerged as a third option, between traditional cultivation and microbial fermentation, and has shown promise for *Astragalus* saponin production in recent years. *Astragalus* hairy roots can be induced by *Agrobacterium rhizogenes* and offer short growth cycles (weeks rather than years), hormone autotrophy, and good genetic stability. Direct injection of *R. rhizogenes* into *A. membranaceus* stems gives induction rates of up to 80.1%, hairy-root biomass peaks at day 28 of culture, and overexpression of *AmUGT15* raised total astragaloside content 4.2-fold over non-transgenic controls [76]. An independent absolute benchmark is available from a 30-L airlift study: after 20 d, hairy roots reached 11.5 g dry biomass/L and 1.4 mg astragaloside IV/g dry weight [112]. Their product is 16.1 mg astragaloside IV/L culture, or an average 0.805 mg/L/day, calculated without adjustment for extraction recovery. These values are separate from the *AmUGT15* fold change, which does not establish an absolute compound-specific yield. Because hairy root culture retains the plant’s metabolic background, it sidesteps the CYP450 heterologous expression bottleneck of yeast while permitting standardized production under controlled conditions. Scale-up still faces engineering challenges, however, including shear sensitivity and inadequate oxygen transfer, and its economics need comparative assessment against yeast fermentation and traditional cultivation.

### 5.7. Future Technology Outlook and Regulatory Considerations

Several technological developments could accelerate progress. The finding that AmOGD1, a 2-oxoglutarate-dependent dioxygenase, participates in triterpenoid backbone modification [32] suggests that other non-CYP450 oxidases may contribute to saponin diversification, given the specific features of the *Astragalus* pathway. Systematic mining of OGD family members could reveal new modifier enzymes. AI-assisted protein structure prediction can accelerate understanding and rational engineering of the catalytic mechanisms of AmCYP88D25, AmCYP88D7, and AmCYP71D756, for instance using molecular docking to predict substrate binding modes and guide directed evolution. The 4 Mb BGC discovered by Xu et al., which lacks a backbone synthase [32], raises a fundamental question: how does *AmOSC3* achieve co-expression with the modifier enzymes inside the cluster? Deciphering the regulatory elements and transcription-factor networks of this BGC would clarify the regulatory logic of *Astragalus* triterpenoid synthesis and supply plant-derived elements for promoter selection in heterologous expression. On the engineering side, scaling from shake flask to industrial fermenter requires solving dissolved-oxygen, mixing-efficiency, and product-inhibition issues. The economics of downstream separation and purification for saponins are equally critical for industrialization [109]. Terpenoid products from microbial fermentation typically require multi-step separation such as solvent extraction, adsorption, chromatography, etc., and in situ extraction combined with two-phase fermentation can reduce product inhibition and simplify downstream processing [109]. Application of synthetic-biology-derived plant natural products in food and pharmaceuticals must be assessed under applicable product, biosafety, and manufacturing-quality requirements. Regulatory treatment differs by jurisdiction and should not be generalized. In the EU, a fermentation-derived saponin intended for food use may require assessment under Regulation (EU) 2015/2283 if within its scope; in the US, food use may be evaluated under applicable food-ingredient provisions, including the GRAS framework (FD&C Act Sections 201(s) and 409) or, for supplements, any applicable new dietary ingredient notification; in China, the applicable food-ingredient or drug-registration requirements depend on intended use, and the production strains may also be subject to applicable biosafety management even though the purified product contains no living engineered organism. Pharmaceutical use additionally triggers GMP requirements. Given this heterogeneity, no claim about approval timelines is made here; regulatory strategy must be developed per market.

## 6. Conclusions and Outlook

The trajectory of *Astragalus* triterpenoid research—genome publication, cluster discovery, enzyme-by-enzyme pathway elucidation, and heterologous reconstitution—offers a transferable template for other medicinal plants. What is established: six representative genome assemblies, a ~4 Mb tailoring-gene cluster, the core route from 2,3-oxidosqualene to astragaloside IV, and proof-of-concept production in yeast (1.04 mg/L cycloastragenol), *N. benthamiana* (2.224 mg astragaloside IV/g dry weight), and engineered hairy roots (4.2-fold total-astragaloside enhancement). What remains unresolved: the in planta downstream route and acetylation steps, transcriptional and chromatin-level regulation, transport and storage, host self-resistance, and donor/cofactor supply—and, on the engineering side, uncertain product-specific productivity and scale-up economics, with de novo astragaloside IV synthesis from simple carbon substrates in yeast not demonstrated in the cited studies. Whether sustainable biomanufacturing is reached will depend on closing these specific, measurable gaps; genome-informed breeding, hairy-root culture, and semi-synthesis provide complementary supply routes in the interim.

## Figures and Tables

**Table 1:** Representative genome assemblies available for *Astragalus* (as of September 2026).

Taxon	Assembly Size	Sequencing Platform	Contig N50	Scaffold N50	Chr. (Anchored)	Gene Number	Completeness (BUSCO/QV)	Assembly Accession	Ref.
*A. mongholicus*	1.47 Gb	PacBio CCS + Illumina + Hi-C	9.79 Mb	181.02 Mb	9 (99.75%)	27,868	BUSCO 96.84%	GPGD 109	[29]
*A. membranaceus* (AM-CLR)	1.43 Gb	PacBio CLR + Illumina + Hi-C	1.67 Mb	184.46 Mb	9 (98.16%)	29,914	BUSCO 97.27%; QV 48.58	GCA_039519185.1	[30,31]
*A. membranaceus* (AM-ONT)	1.44 Gb	MGI short reads + ONT + Hi-C	2.82 Mb	184.69 Mb	9	38,398	BUSCO 92.44%	GCA_042924565.1	[31,32]
*A. membranaceus* (AM-T2T)	1.39 Gb	PacBio HiFi + ultra-long ONT + Hi-C	180.45 Mb	180.45 Mb	8 (T2T)	32,600	BUSCO 99.63%; QV 57.51	GigaDB 10.5524/102751	[31]
*A. sinicus*	595.52 Mb	PacBio + Illumina + Hi-C	1.50 Mb	78.42 Mb	8 (96.66%)	34,253	BUSCO 91.10%	n.r. (see note)	[45]
*A. alpinus* (haplotype 1)	766.58 Mb	PacBio HiFi + Hi-C	1.78 Mb	95.36 Mb	8 (98.55%)	n.r.	BUSCO 96.8%; QV 59.3	GCA_964188245.1	[46]

Note: n.r., not reported. Data records distinguish assembly accessions from BioProjects, raw-read accessions, and dataset DOIs; a BioProject or raw-read identifier is not an assembly accession. The *A. alpinus* haplotype-1 assembly accession is GCA_964188245.1 [46]. AM-ONT genome BUSCO completeness is 92.44% in the original supplementary table of [32], whereas the comparative analysis of [31] reports 93.37%; BUSCO results can differ with software and lineage datasets. Table 1 data records: *A. mongholicus*, GPGD species/109 (assembly record); AM-CLR, GCA_039519185.1 (assembly), PRJNA1067739 (BioProject); AM-ONT, GCA_042924565.1 (assembly), PRJNA917072 (BioProject); AM-T2T, GigaDB DOI 10.5524/102751 (assembly/annotation), SRR35052312 and ERR15401760 (raw reads); *A. sinicus*, PRJNA681368 (BioProject; assembly accession n.r. in source); *A. alpinus* haplotype 1, GCA_964188245.1 (assembly), PRJEB73944 (BioProject). The *A. membranaceus* assembly of [30] and the T2T assembly of [31] originate from material described as *A. membranaceus*, and their chromosome counts (9 vs. 8) remain to be reconciled.

**Table 2:** Summary of key enzymes in *Astragalus* triterpenoid saponin biosynthesis.

Enzyme	Gene/Family	Catalytic Function	Substrate → Product	Reference
AmOSC3/CAS (cycloartenol synthase)	OSC family	2,3-oxidosqualene cyclization	2,3-oxidosqualene → cycloartenol	[29,32,35]
bAS (β-amyrin synthase)	OSC family	2,3-oxidosqualene cyclization	2,3-oxidosqualene → β-amyrin	[29,65]
AmCYP88D25	CYP88D subfamily	C-16 hydroxylation	cycloartenol → 16-hydroxycycloartenol	[32]
AmCYP88D7	CYP88D subfamily	C-6 hydroxylation	16-hydroxycycloartenol → 6,16-dihydroxycycloartenol	[32,67]
AmCYP71D756	CYP71D subfamily	C-24,25 epoxidation	6,16-dihydroxycycloartenol → 24,25-epoxy-6,16-dihydroxycycloartenol	[32]
AmOGD1	2-oxoglutarate-dependent dioxygenase	C-20 hydroxylation	24,25-epoxy intermediate → cycloastragenol (20,24-tetrahydrofuran ring formation is proposed to be spontaneous)	[32]
AmHSD1	hydroxysteroid dehydrogenase	C-3 oxidation (step 1) and C-3 reduction (step 3)	cycloastragenol → cycloastragenol-3-one; cycloastragenol-3-one-6-O-glucoside → cycloastragenol-6-O-glucoside	[33]
AmGT8; AmGT36 (see Table 3)	UGT family	6-O-glucosylation (step 2 in the AmHSD1 route; direct reaction by AmGT36)	AmGT8: cycloastragenol-3-one → cycloastragenol-3-one-6-O-glucoside; AmGT36: astramembrannin II → astragaloside IV	[32,33,34]
AmGT1; AmGT11 (see Table 3)	UGT family	3-O-xylosylation (step 4 in the AmHSD1 route; direct reaction by AmGT11)	AmGT1: cycloastragenol-6-O-glucoside → astragaloside IV; AmGT11: cycloastragenol → astramembrannin II	[32,33,34]
AmGT5/AmGT9	UGT family	glycosylation (multiple substrates)	catalyzes formation of multiple astragalosides	[34]
HMGR	MVA pathway rate-limiting enzyme	HMG-CoA reduction	HMG-CoA → MVA	[29]
SQS/SQE	squalene synthase/epoxidase	squalene synthesis and epoxidation	FPP → squalene → 2,3-oxidosqualene	[29]

**Table 3:** Concordance of gene names for the downstream glycosylation routes.

Function	Zhang et al. [33]/Chen et al. [34]	Xu et al. [32]	Substrate	Product	Assembly/Source	Notes
6-O-glucosylation	*AmGT8*	*AmGT36*	cycloastragenol-3-one (*AmGT8*); cycloastragenol or astramembrannin II (*AmGT36*)	cycloastragenol-3-one 6-O-glucoside (AmGT8); brachyoside B or astragaloside IV, respectively (AmGT36)	AmGT8 (= *UGT73P28*) cloned from *A. membranaceus* [33,34]; AmGT36 (evm.model.LG01.4084; GenBank OQ365045) inside the ~4 Mb BGC (chr1: 117,864,798–121,948,401 bp) of the Xu et al. assembly [32]	Study-specific names; pairwise sequence identity and molecular alias status have not been independently established. Astragaloside III has a 2′-O-glucosylated 3-O-xylosyl chain, unlike brachyoside B [34,35].
3-O-xylosylation	*AmGT1*	*AmGT11*	cycloastragenol 6-O-glucoside (*AmGT1*); cycloastragenol (*AmGT11*)	astragaloside IV (AmGT1); astramembrannin II, the cycloastragenol 3-O-xyloside (AmGT11)	AmGT1 (= *UGT73F41*, GenBank ON075039) cloned in [34]; AmGT11 (evm.model.LG01.4093) inside the ~4 Mb BGC (chr1: 117,864,798–121,948,401 bp) of the Xu et al. assembly [32]	AmGT1 shows donor promiscuity (UDP-Glc/UDP-Xyl) [34]. Pairwise sequence identity, molecular alias status, and a standalone AmGT11 accession have not been independently verified.
C-3 oxidation/reduction	*AmHSD1*	— (not used)	cycloastragenol; cycloastragenol-3-one 6-O-glucoside	cycloastragenol-3-one; cycloastragenol 6-O-glucoside	*A. membranaceus* [33]	Bifunctional hydroxysteroid dehydrogenase; unique to the four-step model

Note: the two enzyme sets have distinct characterized reaction sequences; equivalence of their gene names as molecular aliases has not been established by sequence comparison, and whether both operate in *Astragalus* roots remains unresolved (Section 3.4).

**Table 4:** Heterologous production of *Astragalus*-related compounds.

Target Product	Chassis Organism	Value	Unit & Basis	Format/Scale/Duration	Key Engineering Strategies	Reference
Cycloastragenol	*S. cerevisiae*	1.04	mg/L culture (volumetric titer)	Shake-flask batch, 120 h; working volume n.r.	Peroxisomal MVA engineering; *AmCAS1* + *AmCYP88D25/7*/*AmCYP71D756* + *AmOGD1*	[77]
Astragaloside IV	*N. benthamiana*	2.224	mg/g leaf dry weight; AS-IV-specific	Transient agroinfiltration; harvest day n.r.	Co-expression of *AmOSC3* + *AtCPR1* + *AmCYP88D25/7*/*AmCYP71D756* + *AmOGD1* + *AmGT11/AmGT36* + *NbtHMGR*	[32]
Astragaloside IV	*N. benthamiana*	qualitative	detected product (no titer reported)	Agroinfiltration, transient expression	Co-expression of *AmHSD1* + *AmGT8* + *AmGT1* (four-step pathway validation)	[33]
13 cycloartane-type saponins	In vitro enzyme catalysis	22.6–98.7	% conversion (not a titer)	100-µL sequential cascade; 37 °C; 2 h per step	Combinatorial UGT system; protein engineering (G146V/S, A394F)	[34]
Calycosin-7-O-glucoside	*S. cerevisiae*	47.6	mg/L culture	15 mL in 125-mL flask; 30 °C, 220 rpm, 72 h	*PlOMT9/MtI3′H/AmUGT2* high-copy expression; sucrose/ethanol medium (PP01)	[97]
Calycosin-7-O-glucoside	*S. cerevisiae*	0.22	mg/L culture	Shake flask	*PlOMT9* copy number optimization; UDP-glucose supply optimization	[96]
Squalene (precursor)	*S. cerevisiae*	55.8	g/L culture	Fed-batch (as reviewed)	Comprehensive MVA pathway metabolic engineering	[86]
β-amyrin (analog)	*S. cerevisiae*	4216.6	mg/L culture	Two-stage fed-batch	ERG7 downregulation/ERG1 stability improvement; two-stage fed-batch fermentation	[85]

Note: values reported on different bases (mg/L culture, mg/g biomass, % conversion, fold change) are not directly comparable, and no platform-performance ranking is implied; where available, scale and duration are quoted from the primary studies. n.r. denotes information not reported or not retrievable in the consulted source. The 2.224 mg/g result is astragaloside IV specifically, reported on a dry-weight basis in the main text of [32]; the harvest day is not reported [32].

**Table 5:** Evidence-based comparison of *Astragalus* saponin production platforms (values from cited sources; qualitative where data are lacking).

Metric	Field Cultivation	Hairy-Root Culture	Transient Expression (*N. benthamiana*)	Yeast Fermentation	Feedstock-Based Semi-Synthesis
Cycle time	3–5 years	20 d [112]; max. biomass day 28 in separate study [76]	Harvest day n.r. for the cited AS-IV result [32]	120 h [77]	Reaction duration n.r. here [110,111]
Product level reached	AS IV ≥ 0.080% *w*/*w* root DW; assay QC limit [79,80]	AS IV: 1.4 mg/g DW; 11.5 g DW/L [112]. Separate total-astragaloside increase: 4.2-fold [76]	2.224 mg AS IV/g dry weight [32]	1.04 mg/L cycloastragenol [77]	96.5% AS-IV-to-CAG conversion, gram scale [110]; 2.54-kg CAG preparation [111]
Arable land	Cropland required; matched inventory n.r.	Culture facility; feedstock land footprint n.r.	Plant growth space; matched inventory n.r.	Fermentation facility; feedstock land footprint n.r.	depends on feedstock source
Water/solvent demand	Irrigation and extraction; matched inventory n.r.	Medium and extraction; matched inventory n.r.	Plant cultivation and extraction; inventory n.r.	Medium and DSP; matched inventory n.r. [109]	acid/enzymatic hydrolysis; solvent DSP
Technology readiness	established	30-L bioreactor demonstration [112]	laboratory	laboratory	Gram-scale enzymatic [110]; kilogram-scale chemical [111]; commercial status not established here

Note: n.r., not reported.

## Data Availability

No new data were created or analyzed in this study. Data sharing is not applicable to this article.

## Supplementary Materials

The following supporting information can be downloaded at: https://www.mdpi.com/article/10.3390/plants15213466/s1, Supplementary File S1: Complete database search strings (as run in PubMed and adapted to the syntax of Web of Science Core Collection and Scopus, with per-block record counts for all four thematic blocks).

## Abbreviations

The following abbreviations are used in this manuscript:

AACT	Acetoacetyl-CoA thiolase
AI	Artificial intelligence
API	Active pharmaceutical ingredient
ARE	Antioxidant response element
BGC	Biosynthetic gene cluster
bAS	β-Amyrin synthase
BUSCO	Benchmarking Universal Single-Copy Orthologs
CAS	Cycloartenol synthase
CCS	Circular consensus sequencing
ChIP-seq	Chromatin immunoprecipitation sequencing
CK	Compound K
CoA	Coenzyme A
CPR	NADPH–cytochrome P450 reductase
CRISPR-Cas9	Clustered regularly interspaced short palindromic repeats–CRISPR-associated protein 9
CYP450	Cytochrome P450 monooxygenase
DBTL	Design–build–test–learn
DMAPP	Dimethylallyl diphosphate
DW	Dry weight
ER	Endoplasmic reticulum
FPP	Farnesyl diphosphate
GMP	Good manufacturing practice
GT	Glycosyltransferase
Hi-C	High-throughput chromosome conformation capture
HMG-CoA	3-Hydroxy-3-methylglutaryl coenzyme A
HMGR	3-Hydroxy-3-methylglutaryl-CoA reductase
HMGS	3-Hydroxy-3-methylglutaryl-CoA synthase
HSD	Hydroxysteroid dehydrogenase
IPP	Isopentenyl diphosphate
LAS	Lanosterol synthase
LTR	Long terminal repeat
LUS	Lupeol synthase
MVA	Mevalonate
NRF2	Nuclear factor erythroid 2-related factor 2
OGD	2-Oxoglutarate-dependent dioxygenase
OSC	2,3-Oxidosqualene cyclase
PSPG	Plant secondary product glycosyltransferase
RNAi	RNA interference
SQE	Squalene epoxidase
SQS	Squalene synthase
UDP	Uridine 5′-diphosphate
UGT	UDP-dependent glycosyltransferase
UV	Ultraviolet
