# Supplementary material for: Biosynthetic Gene Clusters of Astragalus Triterpenoid Saponins: From Genome Discovery to Synthetic Biology and Biomanufacturing Prospects
Source: Plants (Basel). 2026 Nov 6;15(21):3466. doi: 10.3390/plants15213466 (PMC13766089; doi:10.3390/plants15213466)
Supplement: Supplementary file 1 [file plants-15-03466-s001.zip › plants-4597135-supplementary.pdf]

Supplementary File S1. Complete database search strings

The PubMed search used a publication-date filter of 2000/01/01–2026/08/01; Web of Science and Scopus were cross-checked in October 2026 with a publication-year filter of 2000–2026. Within each database, the four thematic blocks were combined with the Boolean operator OR, and the resulting record sets were merged and deduplicated. Field tags: [tiab] = title/abstract (PubMed); TS = topic (Web of Science); TITLE-ABS-KEY = title, abstract, keywords (Scopus). Google Scholar was screened with the same term combinations to identify additional relevant literature and was not used for quantitative record counts.

Database	Thematic block	Search string
PubMed	Block 1: Genomics	Astragalus[tiab] AND (genome[tiab] OR genomic[tiab] OR transcriptome*[tiab] OR "whole genome"[tiab])
PubMed	Block 2: Biosynthesis and biosynthetic gene clusters	Astragalus[tiab] AND (triterpenoid*[tiab] OR triterpene*[tiab] OR saponin*[tiab] OR astragaloside*[tiab] OR cycloastragenol[tiab]) AND (biosynthes*[tiab] OR "biosynthetic gene cluster"[tiab] OR "gene cluster"[tiab] OR "cytochrome P450"[tiab] OR glycosyltransferase*[tiab] OR "oxidosqualene cyclase"[tiab] OR pathway[tiab])
PubMed	Block 3: Regulation	Astragalus[tiab] AND (triterpenoid*[tiab] OR triterpene*[tiab] OR saponin*[tiab] OR astragaloside*[tiab]) AND (regulation[tiab] OR "transcription factor"[tiab] OR epigenetic*[tiab] OR chromatin[tiab] OR "gene expression"[tiab])
PubMed	Block 4: Biotechnological production	(Astragalus[tiab] OR astragaloside[tiab] OR cycloastragenol[tiab]) AND ("synthetic biology"[tiab] OR "metabolic engineering"[tiab] OR "heterologous expression"[tiab] OR "heterologous production"[tiab] OR "de novo biosynthesis"[tiab] OR "hairy root"[tiab] OR "microbial production"[tiab] OR "cell factory"[tiab] OR yeast[tiab])
Web of Science (Core Collection)	Block 1: Genomics	TS=(Astragalus AND (genome OR genomic OR transcriptome* OR "whole genome"))
Web of Science (Core Collection)	Block 2: Biosynthesis and biosynthetic gene clusters	TS=(Astragalus AND (triterpenoid* OR triterpene* OR saponin* OR astragaloside* OR cycloastragenol) AND (biosynthes* OR "biosynthetic

		gene cluster" OR "gene cluster" OR "cytochrome P450" OR glycosyltransferase* OR "oxidosqualene cyclase" OR pathway))
Web of Science (Core Collection)	Block 3: Regulation	TS=(Astragalus AND (triterpenoid* OR triterpene* OR saponin* OR astragaloside*) AND (regulation OR "transcription factor*" OR epigenetic* OR chromatin OR "gene expression"))
Web of Science (Core Collection)	Block 4: Biotechnological production	TS=((Astragalus OR astragaloside OR cycloastragenol) AND ("synthetic biology" OR "metabolic engineering" OR "heterologous expression" OR "heterologous production" OR "de novo biosynthesis" OR "hairy root" OR "microbial production" OR "cell factory" OR yeast))
Scopus	Block 1: Genomics	TITLE-ABS-KEY(Astragalus AND (genome OR genomic OR transcriptome* OR "whole genome"))
Scopus	Block 2: Biosynthesis and biosynthetic gene clusters	TITLE-ABS-KEY(Astragalus AND (triterpenoid* OR triterpene* OR saponin* OR astragaloside* OR cycloastragenol) AND (biosynthes* OR "biosynthetic gene cluster" OR "gene cluster" OR "cytochrome P450" OR glycosyltransferase* OR "oxidosqualene cyclase" OR pathway))
Scopus	Block 3: Regulation	TITLE-ABS-KEY(Astragalus AND (triterpenoid* OR triterpene* OR saponin* OR astragaloside*) AND (regulation OR "transcription factor*" OR epigenetic* OR chromatin OR "gene expression"))
Scopus	Block 4: Biotechnological production	TITLE-ABS-KEY((Astragalus OR astragaloside OR cycloastragenol) AND ("synthetic biology" OR "metabolic engineering" OR "heterologous expression" OR "heterologous production" OR "de novo biosynthesis" OR "hairy root" OR "microbial production" OR "cell factory" OR yeast))

PubMed records were limited to publication dates 2000/01/01–2026/08/01. The four thematic blocks retrieved 182, 298, 163, and 58 records in PubMed, respectively (592 unique records after merging). The Web of Science and Scopus cross-checks retrieved 375, 600, 269, and 127 records per block in Web of Science (1,139 unique records after merging) and 652, 800, 706, and 149 records per block in Scopus (1,764 unique records after merging).
